# High uric acid level increases the risk of acute kidney injury in acute coronary syndrome patients

**DOI:** 10.22088/cjim.12.3.323

**Published:** 2021-04

**Authors:** Erny Puti, Haerani Rasyid, Pendrik Tandean, Himawan Sanusi, Hasyim Kasim, Syakib Bakri, Makbul Aman, Arifin Seweng

**Affiliations:** 1 Division of Nephrology, Faculty of Public Health, Hasanuddin University, Makassar, South Sulawesi, Indonesia; 2 Division of Cardiology, Faculty of Public Health, Hasanuddin University, Makassar, South Sulawesi, Indonesia; 3 Division of Endocrine Metabolic and Diabetes, Department of Internal Medicine, Faculty of Medicine, Hasanuddin University, Makassar, South Sulawesi, Indonesia; 4 Biostatistics Department, Faculty of Public Health, Hasanuddin University, Makassar, South Sulawesi, Indonesia

**Keywords:** Acute kidney injury, Acute coronary syndrome, Uric acid

## Abstract

**Background:**

Both clinical and experimental evidence have been published over the past few decades supporting the existence of a close relationship between the elevated levels of serum uric acid with cardiovascular events and acute kidney injury (AKI). This study aimed to determine the effect of serum uric acid levels on the incidence of AKI in acute coronary syndrome (ACS) patients.

**Methods:**

A retrospective cohort study with a cross sectional design was performed. The research was conducted at Dr. Wahidin Sudirohusodo Hospital from October 2019 to December 2019. Nonrandom sampling was employed in the medical records. All patients who met the inclusion criteria were at > 18 years old and diagnosed with ACS with AKI. The demographic data of age, sex and serum uric acid levels were recorded. The data obtained were analyzed using the SPSS (Statistical Package for Social Sciences).

**Results:**

There were 158 subjects of ACS patients with AKI and 135 without AKI. There was a significant correlation between high uric acid levels with the incidence of AKI in ACS (p<0.001). Patients with high serum uric acid levels were 9.5 times at risk of developing AKI compared to those with normal serum uric acid levels.

**Conclusion:**

High uric acid level is one of the risk factors for AKI in ACS and indicates 9.5 times at risk of developing AKI compared to normal serum uric acid level. Therefore, it is necessary to monitor serum uric acid level and kidney function in ACS patients.

Acute coronary syndrome (ACS) is a major cardiovascular problem because it causes high rates of hospital care and mortality. Most of the ACS is an acute manifestation of coronary blood vessel atheroma plaque, that ruptures due to changes in the composition of plaque (1). AKI is a complication that commonly occurs in patients with ACS associated with high mortality rates and length of stay in hospital (2). It is a complex disorder characterized by early worsening (usually within hours or days) of renal function and exhibits clinical manifestations ranging from minimal increase in creatinine serum to anuric that can be provoked by various medical conditions (3). The incidence of acute kidney injury has increased over 30% in ACS patients and is associated with higher short- and long-term morbidity and mortality (4). Nevertheless, the risk factors for AKI and its consequences in patients with AMI are still unclear (5). Uric acid (UA) is the eventual product of purine metabolism which is processed by xanthine oxidase (6). Clinical and experimental evidence has been brought out over the past few decades, confirming the association of increased levels of serum uric acid (SUA) with hypertension, metabolic diseases, chronic kidney disease, acute kidney injury and cardiovascular events (7).

Xianlian Xu et al. in meta-analysis study assessing the correlation of serum uric acid levels with the incidence of AKI in 18 cohort studies found that the increased uric acid levels raised the risk of AKI in patients (8). In adition, Min wo Kang et al. also reported that hyperuricemia increases the risks of AKI and all cause mortality in hospital patients (9). Cheungpasitporn W et al. reported that there was an association between elevated admission of serum uric acid and an increased risk for hospital AKI (10). No previous studies that assess the effect of uric acid on the incidence of AKI in ACS patient have been found. Therefore, we conduct the research to provide sufficient information for this topic.

## Methods

A retrospective cohort study with a cross-sectional design was performed. The data were collected at Dr. Wahidin Sudirohusodo General Hospital from October to December 2019. The study population was the ACS patients with AKI who met the inclusion criteria. The inclusion criteria were the patients at the age of >18 years, diagnosed ACS with AKI, who completed medical record data. The exclusion criteria were those with severe infections, chronic kidney disease, urinary tract obstruction, and hypovolemic shock, taking lipid-lowering drugs and undergoing percutaneous coronary intervention (PCI). Diagnosis of ACS was based on history taking, laboratory examination, and electrocardiography (ECG) examination. ACS was divided into acute myocardial infarction with ST segment elevation (STEMI), acute myocardial infarction without ST segment elevation (NSTEMI), and unstable angina pectoris (UAP). 

The diagnosis of STEMI was established if there was a typical angina complaint with a persistent ECG elevation of ST segments in two adjacent leads or a new bundle branch block. The diagnosis of NSTEMI was determined if there was a typical angina complaint with ECG images in the form of ST segment depression, T wave inversion, or flat T waves without ST segment elevation, and increased cardiac biomarker examination. The diagnosis of UAP was determined if there were complaints of acute angina pectoris with ECG images in the form of ST segment depression, T wave inversion, or flat T waves without ST segment elevation images, and normal cardiac biomarker examination results. The diagnosis of AKI was based on the 2012 KDIGO criteria, an increase in serum creatinine by ≥ 0,3 mg/dl within 48 hours, or increase in serum creatinine to ≥ 1,5 times *baseline* which is known or presumed to have occurred within the prior 7 days, or urine volume ≤ 0.5ml/kg/h for 6 hours. Assessment of uric acid was reported in quantitative form (mg/dl) and categorized high if uric acid serum remained at >7.0 mg/dl in male and >5.7 mg/dl in female respectively.

Nonrandom sampling in medical records was performed. All medical records of patients diagnosed with ACS with AKI from January 2017 to January 2019, which met the inclusion criteria, were collected. Afterwards, the data (age, sex, serum uric acid level) were recorded. The SPSS Version 22 was employed to analyze the data. The statistical analysis was descriptive statistical calculations and frequency distribution as well as the independent-t statistical test, chi square test and the calculation of odds ratio (OR) values. The test results were considered significant by a p-value of <0.05. This study was permitted and acknowledged by Hasanuddin University Ethics Medical Committee, with a reference number: 487/UN4.6.4.5.3.1/PP36/2019.

## Results

Data analysis was performed among 158 ACS subjects with AKI and 135 ACS without AKI patients. The average age was 64.3 years old in ACS patients with AKI and 61.2 years in without AKI patients. The majority of research subjects in both groups was male (78.5%) in ACS with AKI and (76.3%) in ACS without AKI respectively. Hyperuricemia was found in 129 (81.6%) subjects in the ACS group with AKI and 43 (43%) subjects in ACS group without AKI. Table 1 shows a significant relationship between high uric acid levels with the incidence of AKI on ACS (p<0.001) and (OR 9.5) with a higher percentage of uric acid in the ACS group with AKI of 81.6% compared to ACS patients without AKI 31.9%. 

**Table 1:** Relationship of sex, age, and uric acid with AKI on ACS

**Variable**		**AKI (n=158)**		**Without AKI (n=135)**		**p**	**OR ** **(95% CI)**
		n	%	n	%		
Gender	Male	124	78,5	103	76,3	0,655	1,1 (0,65-1,96)
	Female	34	21,5	32	23,7		
Age	18-65 years old	87	55,1	89	65,9	0,058	0,6 (0,39-1,02)
	>65 years old	71	44,9	46	34,1		
Uric Acid	High	129	81,6	43	31,9	0,000	9,5 (5,54-16,36)
	Normal	29	18,4	92	68,1		

**Figure 1:**
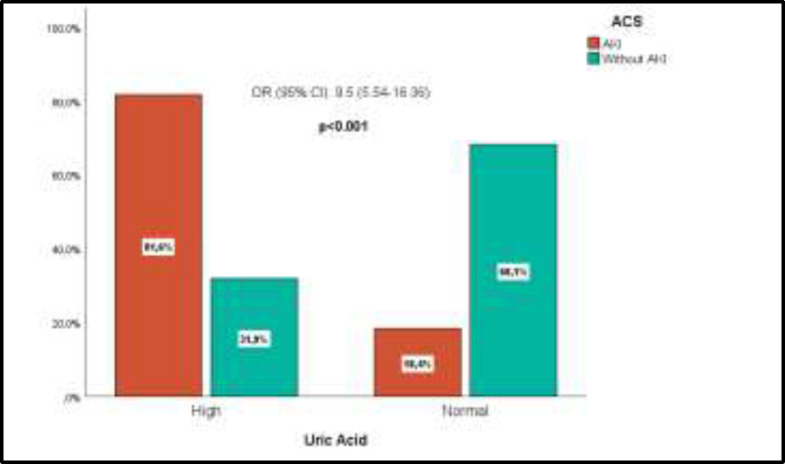
The proportion of AKI by uric acid level

Based on the age and gender, there was no significant relationship to the incidence of AKI in ACS. Figure 1 shows the proportion of AKI according to uric acid level, in which the uric acid levels were higher in ACS patients with AKI (81,6%) compared to ACS patients without AKI (31,9%).

## Discussion

Previous studies have determined the association between hyperuricemia and the risk of AKI under various pathological conditions (9). The study conducted by Wisit C et al. also revealed a significant relationship between increased SUA and the incidence of AKI in the hospital (10). Another study by Kazunori Otomo et al. also found that hyperuricemia became a risk factor of the occurrence of AKI in preoperative patients and those treated at the hospital (11). Ejazz AA et al. also reported that uric acid might be a novel risk factor of AKI in patients undergoing a high-risk cardiovascular surgery (12). Kim JH et al. also revealed that the baseline of SUA level might be a proper clinical marker for the patients who were at risk of mortality and AKI after acute paraquat intoxication (13). This study reveals that there is a significant relationship between high serum uric acid levels and the incidence of AKI in ACS patients (p<0.001). In addition, the patients with high serum uric acid levels are 9.5 times at risk of developing AKI when compared with those with normal SUA levels. Another study suggests that increased SUA level is also associated with several diseases such as myocardial infarction and atherosclerosis, both of which are also the most frequent risk factors for AKI (8). This result supports the results of our study where the risk of AKI is higher in ACS patients with high serum uric acid levels compared to normal uric acid. The mechanism of the occurrence of AKI in ACS patients is very complex, including hemodynamic disorders, activation of the sympathetic nervous system and renin angiotensin aldosterone (RAAS), humoral signaling and inflammation are very important roles in the incidence of AKI (14). These mechanisms are also found in the pathological mechanism that connects uric acid to AKI events. Uric acid induces renal vasoconstriction, impairs autoregulation and hyperuricemia, and stimulates vascular smooth muscle cell proliferation and migration. Also, it causes thickening of the preglomerular arterioles and impairs renal blood flow autoregulation. Hyperuricemia has been proven to upregulate the expression of angiotensinogen, angiotensin-converting enzyme, and angiotensin II receptors and increase angiotensin II levels. In fact, angiotensin II also activates nicotinamide adenine dinucleotide phosphate oxidase (NADPH). Uric acid also stimulates the expression of proinflammatory molecules monocyte chemoattractant protein-1 (MCP-1), C-reactive protein, and generation of oxidants and peroxynitrite. It can stimulate apoptosis through caspase activation or lead to necrosis by peroxidation and nitration. Furthermore, it also induces mitochondrial and endothelial dysfunction through the activation of angiotensin II which causes the activation of endothelial cell NADPH oxidase and the formation of peroxinitrite (15, 16). The relationship between the two mechanisms supports our results that high serum uric acid levels in ACS increases the risk of AKI.

However, hyperuricemia may be present at up to 20% in some populations. Alcohol, purine rich food, and drugs also play an important role in the pathogenesis of hyperuricemia (17). Study reported by Paulus found that drugs were a major factor in the development of an elevated serum uric acid concentration in up to 20% of the hyperuricaemic subjects in one hospital study (18). Choi HK et al. found that the effect of individual alcoholic beverages on uric acid levels varied substantially; beer conferred a larger increase than liquor whereas moderate wine drinking did not increase serum uric acid level (19). 

In this study, the history of drug use, alcohol and types of food consumed was not included in the exclusion criteria because we did not have complete data. In fact, this is a limitation in our study. In addition, we did not assess other laboratory data that may influence the incidence of AKI in ACS. In fact, the studies on the effect of uric acid level on the incidence of AKI on ACS are still lacking, and the sample used in this study is expected to be sufficient. Further and more detailed researches are still needed regarding other cardiovascular risk factors that influence the incidence of AKI in ACS, and it is necessary to monitor the uric acid and kidney function regularly in ACS patients. In Conclusion, this study reveals that high serum uric acid levels are the risk factor for AKI in ACS, and the subjects are 9.5 times at risk of developing AKI compared to those with normal serum uric acid levels. Therefore, it is necessary to monitor the serum uric acid level and kidney function in ACS patients.
